# Peritoneal Modulators of EZH2-miR-155 Cross-Talk in Endometriosis

**DOI:** 10.3390/ijms22073492

**Published:** 2021-03-28

**Authors:** Sarah Brunty, Kristeena Ray Wright, Brenda Mitchell, Nalini Santanam

**Affiliations:** 1 Department of Biomedical Sciences, Joan C. Edwards School of Medicine, Marshall University, Huntington, WV 25755, USA; binion2@live.marshall.edu (S.B.); kristeena.ray@gmail.com (K.R.W.); 2 Department of Obstetrics and Gynecology, Joan C. Edwards School of Medicine, Marshall University, Huntington, WV 25755, USA; dawleyb@marshall.edu

**Keywords:** endometriosis, epigenetics, EZH2, microRNA

## Abstract

Activation of trimethylation of histone 3 lysine 27 (H3K27me3) by EZH2, a component of the Polycomb repressive complex 2 (PRC2), is suggested to play a role in endometriosis. However, the mechanism by which this complex is dysregulated in endometriosis is not completely understood. Here, using eutopic and ectopic tissues, as well as peritoneal fluid (PF) from IRB-approved and consented patients with and without endometriosis, the expression of PRC2 complex components, JARID2, miR-155 (known regulators of EZH2), and a key inflammatory modulator, FOXP3, was measured. A higher expression of EZH2, H3K27me3, JARID2, and FOXP3 as well as miR-155 was noted in both the patient tissues and in endometrial PF treated cells. Gain-or-loss of function of miR-155 showed an effect on the PRC2 complex but had little effect on JARID2 expression, suggesting alternate pathways. Chromatin immunoprecipitation followed by qPCR showed differential expression of PRC2 complex proteins and its associated binding partners in JARID2 vs. EZH2 pull down assays. In particular, endometriotic PF treatment increased the expression of *PHF19* (*p* = 0.0474), a gene silencer and co-factor that promotes PRC2 interaction with its targets. Thus, these studies have identified the potential novel crosstalk between miR-155-PRC2 complex-JARID2 and PHF19 in endometriosis, providing an opportunity to test other epigenetic targets in endometriosis.

## 1. Introduction

Endometriosis is defined by the presence of endometrial tissue in ectopic locations, typically in or around the peritoneal cavity [1,2]. While the exact prevalence of endometriosis is likely underrepresented, most sources cite that a minimum of 10% of women in their reproductive years have this disease [3,4,5]. Primarily described as a hormonal disorder, the pathogenesis of endometriosis has also been linked to immunological/inflammatory, genetic, and environmental factors. More recently, the role of epigenetics in the development and progression of this disorder has been investigated [6,7,8,9,10,11,12]. Epigenetic mechanisms are heritable changes to one’s phenotype that are not associated with a change in nucleotide sequence and include DNA methylation, post-translational modifications to histone proteins, and often microRNAs [13,14].

In addition to heterochromatin-like protein 1 (HP-1), polycomb (PcG) and trithorax (TrxG) complexes are at the heart of epigenetics. Responsible for maintaining gene repression and activity, respectively [15], the latter two complexes function antagonistically to establish epigenetic regulation [15]. Polycomb repressive complex 1 (PRC1), polycomb repressive complex 2 (PRC2), and Pho repressive complex (PhoRC) all form the PcG complexes, with the former two typically being the subject of extensive epigenetic research. The Polycomb Repressive Complex 2 (PRC2) consists of four core proteins, RbAp46/48, Embryonic Ectoderm Development (EED), Suppressor of Zeste 12 (SUZ12), and Enhancer of Zeste Homolog 2 (EZH2), the catalytic subunit of the PRC2 complex. These components work together to regulate chromatin structure via tri-methylation of lysine 27 on histone 3 (H3K27me3) [16,17], which is also known to interact with PRC1. EED binds the histone site while EZH2 methylates it, with the help of SUZ12 [18]. This modification leads to the formation of closed chromatin structure (heterochromatin) and thus marks transcriptional repression, as further demonstrated by the presence of other co-factors [19,20,21]. 

There is very little known about the mechanistic role of PRC2 complex and how it is regulated in the endometriosis disease process. While an in vivo study showed heightened expression of EZH2 and trimethylation of H3K27 in secretory endometrium and endometriotic lesions [22,23], another cell culture study showed that inhibition of PGE2 receptors EP3 and EP4 occur concurrently with decreased EZH2 expression [24], supporting a role for PRC2 in endometriosis-associated pain. 

It has been shown that the PRC2 complex (specifically EZH2) is, at least partly, regulated by Jumonji and AT-Rich Interaction Domain Containing 2 (JARID2) [25], a member of the largest family of histone demethylases, the jumonji family, where all but JARID2 contain the catalytic JmjC domain responsible for histone demethylation [26,27]. Research has found that JARID2 is a cofactor for PRC2 [28]. Additionally, its methylation by the PRC2 complex at K116 is part of a regulatory mechanism that controls the PRC2 enzymatic activity where the methylated JARID2 binds to the EED component of the PRC2 complex. This is required for efficient deposition of H3K27me3 during cell differentiation and fine-tunes the PRC2 activity [29]. JARDI2 is thought to be crucial in the development and progression of cancer. This is due to its cross-talk with EZH2 and PRC2 activity in embryonic stem cells (ESC), as JARID2 is necessary for proper ESC differentiation [25,30,31].

JARID2 is suggested to be modulated by few mechanisms. For example, iron oxidation, which occurs due to increased reactive oxygen species generation and known to be present in excess in women with endometriosis, blocks the catalytic activity of JARID2 [32]. JARID2 is also a common target of microRNAs some of which have been identified by our laboratory to be differentially expressed (miR-30b, miR-30c, miR-10a, miR-29a, miR-26a, miR-148a, miR-181a, miR-30e) in endometriotic lesions compared to control tissues [33]. Palma et al. showed in acute lymphoid leukemia that miR-155-5p induced cell death via a network of mechanisms, including regulation of cyclinD1 by JARID2 [34]. Other such studies support the possibility that miR-155-5p could have been evolved to regulate PRC2 by tweaking JARID2 expression [35]. Interestingly, miR-155-5p is an established promoter of inflammation via regulation of macrophages and cytokines [35,36,37]. Thus, targeting this demethylase (JARID2) via modulators such as microRNAs, could be a novel method of treatment for endometriosis. 

miR-155 is highly expressed in regulatory T-cells (Tregs), where it is targeted by transcription factor forkhead box P3 (FOXP3) [38]. Though limited in evidence, FOXP3 also plays a role in the inflammatory aspect of endometriosis, which correlates with miR-155-5p being a promoter of inflammation. The prevalence of FOXP3^+^ Tregs in an endometriotic environment during secretory phase prevent leukocyte recruitment to the sites of endometriosis [39]. Additionally, peritoneal fluid (PF) from women with endometriosis has a higher concentration of FOXP3-expressing TCD4^+^CD25^high^ cells than the PF of control patients [40,41]. Other studies have also shown that FoxP3 is an inducer of miR-155 [42].

It is important to note that FOXP3 also has an indirect relationship with the EZH2 component of PRC2. Overexpression of the FOXP3 protein not only lessened the proliferative effects of EZH2, but also enhanced degradation of the EZH2 protein in breast cancer models [43]. Conversely, there is evidence that trimethylation of H3K27 by EZH2 is capable of silencing FOXP3 promoter regions, therefore leading to aberrant Treg cell differentiation and function [44]. These studies suggest a complex interplay between epigenetic mediators, PRC2 complex, miR-155-5p, JARID2 and the inflammatory mediator FOXP3. In this study, it is hypothesized that the imbalance in this crosstalk triggers inflammatory responses and possibly nociception in endometriosis. This current study investigated the crosstalk between these mediators in endometriotic patient tissues and in an endometriosis cell model.

## 2. Results

### 2.1. PRC2 Complex and JARID2 mRNA and Protein Expression in Endometriotic Tissues

**Figure 1:**
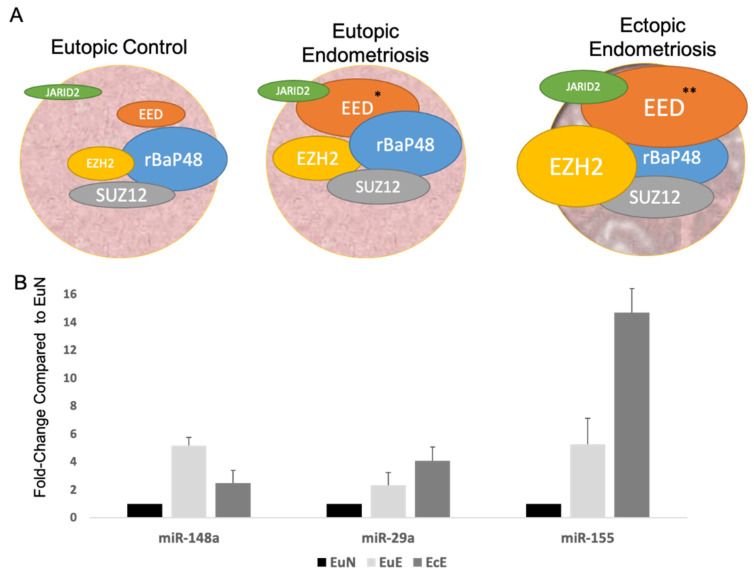
mRNA expression of PRC2 complex and JARID2 and miRNAs that target JARID2 in endometriotic tissues. (**A**) Relative mRNA expression of polycomb repressor complex 2 (PRC2) elements and *JARID2* in eutopic tissues from control women, EuN (*n* = 5), or eutopic and ectopic tissues from women with endometriosis, EuE (*n* = 10) and EcE tissues (*n* = 6). In general, these elements were upregulated in both eutopic and ectopic endo tissues compared to control tissue with *EED* showing significant upregulation in both the eutopic (*p* = 0.0153) and ectopic (*p* = 0.0067). *JARID2* expression was higher in EcE. * *p* < 0.05, ** *p* < 0.01 when compared to EuN tissues. (**B**) Compared to control tissues (*n* = 7), expression of miR-148a, miR-29a, and miR-155 (miRNAs that target JARID2) were all higher in endo tissues (both eutopic and ectopic, *n* = 8).

The endogenous expression of PRC2 complex proteins in endometriotic tissues were first determined. qPCR was used to determine the mRNA expression of PRC2 components SUZ12, EED, and EZH2 in eutopic tissue from women with no endometriosis (EuN, *n* = 5) or women with endometriosis (EuE, *n* = 10) and ectopic tissue from women with endometriosis (EcE, *n* = 6) (Figure 1A). When compared to the EuN tissues, expression of all three PRC2 protein complex (*SUZ12*, *EED* and *EZH2*) and *JARID2*, was higher in both the eutopic (EuE) and ectopic (EcE) tissue from endometriosis patients. Compared to EuN tissues, *SUZ12* levels increased close to 2-fold for EcE but was not significant, however there was a significant increase in *EED* expression by 5.07-fold in EuE (*p* = 0.0153) and 7.13-fold (*p* = 0.0067) in EcE. *EZH2* expression was also increased 2.35-fold in the EuE and 3.10-fold in the EcE but did not reach significance. Expression for *JARID2* increased over 2-fold in EcE tissues, but this was not significant. 

Protein expression was also determined using the automated Western blotting system, WES. While EZH2 showed a significant increase of >7 fold (*p* = 0.0219) in EcE tissues compared to EuN, no significant difference was seen in expression of H3K27me3 or JARID2 (Figure S1). This lack of change in JARID2 expression might be attributed to its altered regulation 

### 2.2. miRNAs Targeting JARID2 in Endometriotic Tissues

The expression levels of miRNAs that regulate JARID2 was next determined in the patient tissues. miRNA qPCR assays were used to measure expression of miR-148a, miR-29a, and miR-155, which, among others, target JARID2 (Targetscan 7.1 and Ingenuity Pathway Analysis Qiagen, Germantown, MD, USA). Interestingly, all three miRNAs were overexpressed in both EuE and EcE tissues compared to EuN tissues (Figure 1B). Both miR-148a and miR-155 showed an over 5-fold increase in expression for the EuE tissues and were also shown to be induced more than 2.5–14-fold, respectively on EcE, while miR-29a expression increased 2–4-fold with levels higher in EuE and EcE tissues. 

### 2.3. PRC2 Complex mRNA and Protein Expression in PF Treated Endometrial Cells

**Figure 2:**
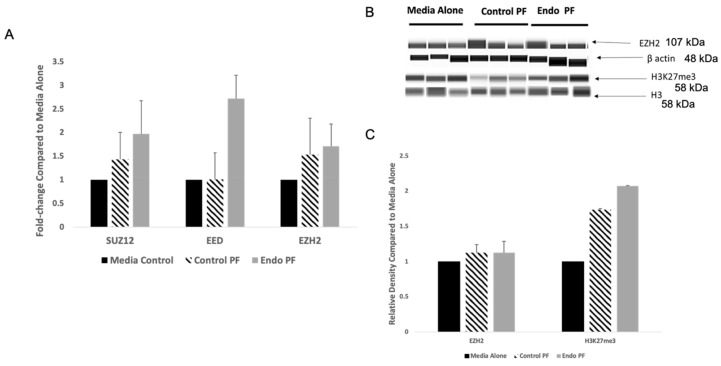
mRNA and protein expression of PRC2 complex proteins in PF treated endometrial cells. (**A**) mRNA expression of *SUZ12*, *EED*, and *EZH2* in cells treated with control PF (*n* = 12) and endo PF (*n* = 13), relative to expression in a media control (*p* > 0.05). (**B**) Representative WES images and densitometric analysis for EZH2, and H3K27me3 in PF-treated cells. **(C**) Relative protein expression of EZH2, and H3K27me3 in PF-treated cells was calculated in relation to a media control and presented as a ratio in which media alone is 1.Densities of protein bands obtained were normalized to β-actin or H3. It is to be noted that molecular weights of protein bands in automated WES system differs from the traditional Western blotting, due to differences in technology.

Peritoneal cavity is one of the major sites for endometriotic lesions in women with endometriosis [45,46]. These patients also exhibit larger volumes of PF rich in inflammatory and nociceptive molecules [47,48]. Current theories propose a dynamic role for PF in modulating the growth of endometriotic lesions, which might be epigenetically regulated by the altered expression of certain miRNAs previously shown in endometriosis [49,50]. Whether PF from patients with and without endometriosis differentially regulated the PRC2 complex proteins in endometrial cells was determined. For this, human endometrial cells were exposed to 1% PF from women with (*n* = 13) or without endometriosis (*n* = 12) for 48 h followed by the measurement of both mRNA and protein expression of PRC2 complex proteins using similar techniques as described for the endometriotic tissues. Cells treated with both 1% control or endo PF had increased *SUZ12*, *EED*, and *EZH2* mRNA expression but none were shown to be statistically significant (Figure 2A). When protein expression was determined using the automated Western Blotting system, WES, EZH2 showed no significant difference in expression levels when compared to the media control. While H3K27me3 did show an upregulation of over 2-fold for endo PF treated cells, this was not significant. (Figure 2B,C).

### 2.4. JARID2 and miRNAs Targeting It in PF Treated Endometrial Cells 

**Figure 3:**
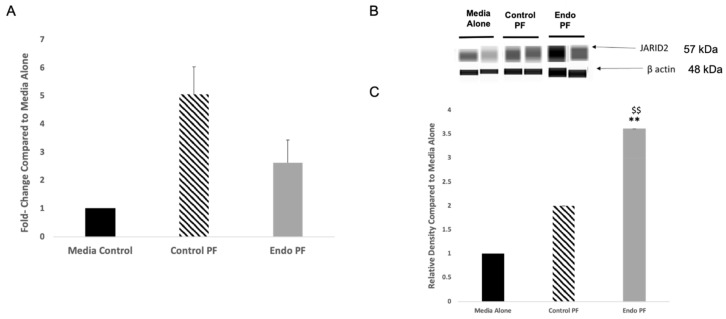
mRNA and protein expression of JARID2 in PF treated endometrial cells. (**A**) mRNA expression of JARID2 in cells treated with control PF (*n* = 12) and endo PF (*n* = 13), relative to expression in a media control (*n* = 6) (*p* > 0.05). (**B**) Representative WES images and densitometric analysis for JARID2 in PF-treated cells. (**C**) Relative protein expression of JARID2 in PF-treated cells was calculated in relation to a media control and presented as a ratio in which media alone is 1. Significant upregulation of JARID2 of 3.61-fold was seen in the endo PF treated cells when compared to media alone cells (*p* = 0.0027) and by about 2-fold compared to control PF (*p* = 0.0096) treated cells. ** significant difference (*p* < 0.01) when compared to media alone. ^$$^ Significant difference (*p* < 0.05) in mean compared to control PF. Densities of the protein bands obtained were normalized to β-actin or H3. It is to be noted that molecular weights of protein bands in automated WES system differs from the traditional Western blotting, due to differences in technology.

The expression of JARID2 in the peritoneal fluid treated endometrial cells was also examined. While both the control and endo PF treated cells showed an increase in mRNA expression of *JARID2* when compared to media alone, neither was shown to be significant (Figure 3A). Analysis of protein expression of JARID2 showed a significant upregulation of expression when cells were treated with endo PF of 3.61-fold (*p* = 0.0027) and by about 2-fold compared to control PF (*p* = 0.0096) treated cells (Figure 3B,C). 

**Figure 4:**
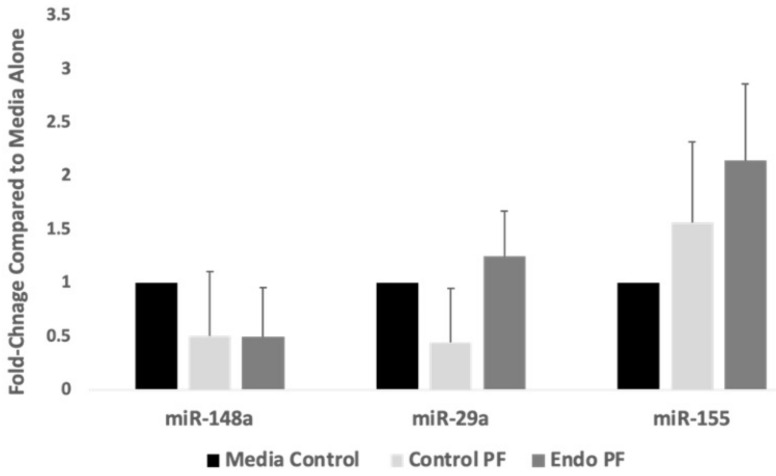
Expression of miRNAs that target JARID2 in PF treated endometrial cells. Compared to media control cells (*n* = 4), expression of miR-148a was shown to be lower in the endo PF treated cells while miR-155 and miR-29a was increased in expression but none were significant. control PF (*n* = 12), endo PF (*n* = 13).

Next, it was assessed if the addition of the PF to the endometrial cells changed the expression levels of the miRNAs that regulates JARID2 levels. miR-148a and miR-29a showed a decrease in expression in both control and endo PF treated endometrial cells, while miR-29a showed a decrease in expression for the control PF treated cells but a slight increase in the endo PF treated cells. Surprisingly, but consistent with what was observed earlier in the EcE tissues, miR-155 showed an increase in expression in both control and endo PF treated cells, but no results were shown to be statistically significant. (Figure 4). This increase in miR-155 might have lowered the JARID2 expression. 

### 2.5. FOXP3 mRNA and Protein Expression in Endometrial Tissues and PF Treated Endometrial Cells

**Figure 5:**
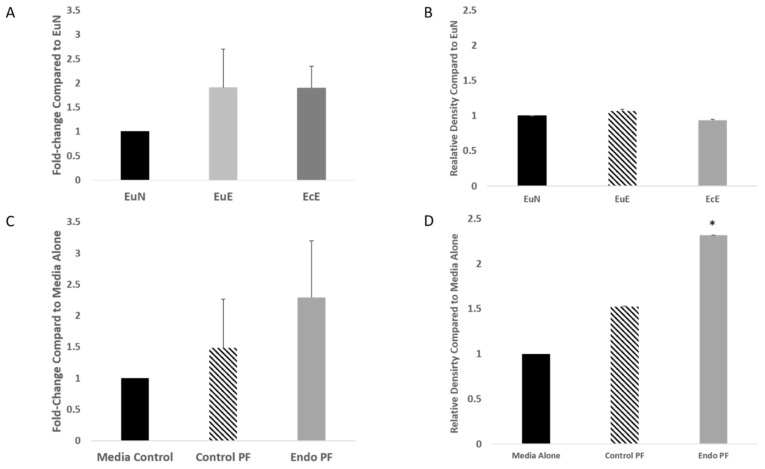
mRNA and protein expression of FOXP3 in patient tissues and PF treated cells. (**A**) Relative mRNA expression of *FOXP3* in EuN (*n* = 5), EuE (*n* = 10), and EcE (*n* = 6). Upregulation of the *FOXP3* mRNA expression in the endometriotic patient tissues was observed but was not significant; (**B**) Relative protein expression of FOXP3 in EuE and EcE was calculated in relation to EuN. Down-regulation of EuE and EcE were seen when compared to EuN. For all comparisons made, *p* > 0.05. (**C**) mRNA expression of FOXP3 in cells treated with control PF (*n* = 12) and endo PF (*n* = 13), relative to expression in a media control (*p* > 0.05). (**D**) Relative protein expression of FOXP3 in PF-treated cells was calculated in relation to a media control and presented as a ratio, in which media alone or EuN is 1. FOXP3 expression was 2.32-fold higher in endo PF (*p* = 0.0493) than in control media alone treated cells. * *p* < 0.05. Densities of the protein bands obtained were normalized to β-actin or H3.

With the knowledge that FOXP3 is a regulator of miR-155, the expression levels of FOXP3 in the endometrial tissues and in the PF treated endometrial cells were determined. qPCR showed that while the tissues from patients with endometriosis (EuE and EcE) were slightly upregulated compared to EuN, there was no significance between the expressions (Figure 5A). Relative protein expressions of FOXP3 are shown in Figure 5B. No significant difference was seen between the mean density of endo tissue and control tissue bands.

*FOXP3* mRNA expression in the endo PF treated cells were shown to be increased in expression but was not significant (Figure 5C). For protein expression using the automated Western blotting system, WES, FOXP3 was increased in cells treated with both control and endo PF, but a statistically significant change in expression was only shown with endo PF treatment (2.32-fold, *p* = 0.0493) (Figure 5D). 

### 2.6. miR-155 Regulates PRC2 Complex and FOXP3 

**Figure 6:**
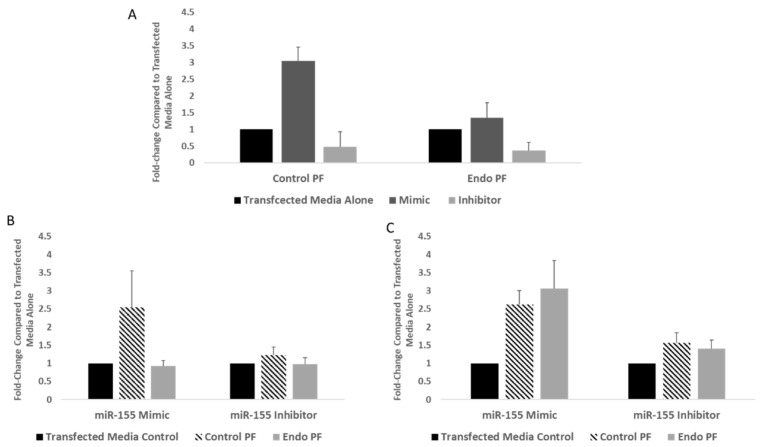
Key mRNA levels in cells transfected with a miR-155 mimic and inhibitor. (**A**) Levels of miR-155 showing transfection efficiency. (**B**) Transfection with a miR-155 mimic had little effect on *JARID2* expression in PF-treated cells (*p* > 0.05), (**C**) but seemed to increase *FOXP3* expression in cells treated with control PF. Compared to control media, the miR-155 inhibitor had no major effect on *JARID2* or *FOXP3* expression in PF treated cells.

Since JARID2 and FOXP3 are targets of miR-155 and miR-155 was upregulated in endometriotic tissues and PF treated cells, it was investigated if modulating miR-155 levels using a mimic or inhibitor will alter these target genes. To test this, the expression of JARID2, PRC2 complex and FOXP3 were determined in endometrial cells transfected with a miR-155 mimic or inhibitor (antagonist). Transfection efficiency of miR-155 is shown in Figure 6A. In cells transfected with the mimic, treatment with control PF increased the expression of miR-155 by over 3-fold, compared to when treated with the inhibitor, where the expression decreased below 0.50-fold. In contrast, in cells transfected with the mimic, treatment with endo PF increased miR-155 expression by 1.5-fold but decreased below 0.50-fold after treatment in cells transfected with the inhibitor. Upon PF treatment of the cells transfected with the miR-155 mimic, there was minimal effect on *JARID2* mRNA expression (*p* > 0.05), but seemed to increase *FOXP3* expression in cells treated with control PF and even more so in cells treated with endo PF. In contrast, PF treatment of miR-155 inhibitor transfected cells had no major effect on *JARID2* or *FOXP3* mRNA expression (Figure 6B,C).

**Figure 7:**
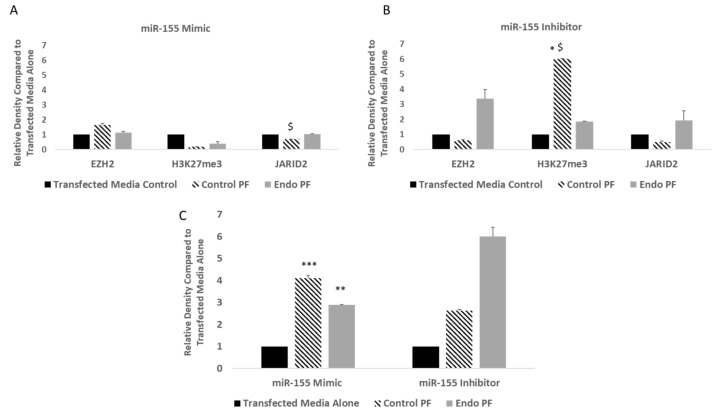
Expression of key targets in cells transfected with miR-155 mimic and inhibitor. (**A**) Transfection with a miR-155 mimic resulted in significantly lower JARID2 expression in control PF-treated cells (*n* = 6) compared to endo PF-treated cells (*n* = 6) (*p* = 0.0106). No significant difference in expression was seen in EZH2 or H3K27me3. (**B**) Transfection with a miR-155 inhibitor resulted in higher H3K27me3 for the control PF-treated cells when compared to transfected media and endo PF-treated cells (*p* = 0.0105, 0.0138, respectively). Relative expression of EZH2 and JARID2 showed no significant increase or decrease in any of the treated groups. (**C**) Transfection with an miR-155 mimic showed significant upregulation of FOXP3 in both control and endo PF-treated cells (*p* = 0.0005, 0.0079) when compared to transfected media alone. Though FOXP3 was induced by 3 or 6-fold in control or endo PF treated cells transfected with miR-155 inhibitor, no significance was observed. * *p* < 0.05, ** *p* < 0.01, and *** *p* < 0.005 compared to transfected media alone, ^$^ Significant difference (*p* < 0.05) in mean compared to endo PF. Density of protein bands obtained was normalized to β-actin or H3.

Western blotting analysis showed that overexpression of miR-155 resulted in significantly lower JARID2 protein expression in control PF-treated cells compared to endo PF-treated cells (*p* = 0.0106). Neither EZH2 nor H2K27me3 protein expression showed any significant up or downregulation in cells overexpressing miR-155 (Figure 7A). In contrast, while both JARID2 and EZH2 showed an upregulation in protein expression in the endo PF treatment groups, when miR-155 was inhibited, no significance was achieved. The protein expression of H3K27me3 in the control PF-treated cells in miR-155 inhibited cells, was significantly upregulated when compared to both the transfected media alone cells and the endo PF-treated cells (*p* = 0.0105 and 0.0138, respectively) (Figure 7B). In miR-155 overexpressing cells, both control and endo PF significantly increased FOXP3 protein expression when compared to transfected media alone (*p* = 0.0005 and 0.0079, respectively). In contrast no significant difference in FOXP3 protein expression was seen in cells transfected with an miR-155 inhibitor (Figure 7C). 

### 2.7. ChIP Using JARID2 or EZH2 Antibody Reveals Other Co-Factors of PRC2 Complex

In order to delineate the alterations in the binding partners of EZH2 in the PF treated cells, ChIP was performed using either JARID2 or EZH2 antibodies followed by ChiP-qPCR promoter array of genes associated with polycomb and trithorax complexes in cells treated with or without PF. Table 1A provides a list of focused gene panel involved in polycomb and trithorax complex activity, that were differentially expressed in endo PF treated cells when compared to control PF after IP by either JARID2 or EZH2 antibodies. The enrichment of EZH2 after JARID2 IP was lower in endo PF-treated cells compared to control cells. However, EZH1, a polycomb enzyme which is responsible for mono-, di-, or tri-methylation of H3K27, showed an enrichment after JARID2 IP in endo treated cells when compared to control PF treated cells but this enrichment was not significant. In contrast, the enrichment of JARID2 after EZH2 IP was over 5-fold higher in endo PF treated cells compared to control PF treated cells. ARID1A, a subunit of the SWI/SNF complex, with an antagonistic relationship with EZH2 [51], showed enrichment after both JARID2 IP and even higher after EZH2 IP. 

When comparing the genes involved in polycomb and trithorax complex activity, pulled down by the two antibodies, (Table 1B), JARID2 IP compared to EZH2 IP showed upregulation of 4 genes with *p*-values < 0.05 in control PF treated cells. While no significant *p*-values were seen for any genes in the endo PF treated cells, when JARID2/EZH2 ratio was calculated, all but 7 genes were shown to be downregulated in these cells suggesting that ChIP by EZH2 in endo PF treated cells has more of an effect on the pull-down expression of these genes compared to JARID2. Any *p*-values > 0.05 may be due in part to the smaller sample size tested. 

### 2.8. PHF19, a Key Co-Factor in the miR-155-JARID2-EZH2 Crosstalk in Endometriosis 

**Figure 8:**
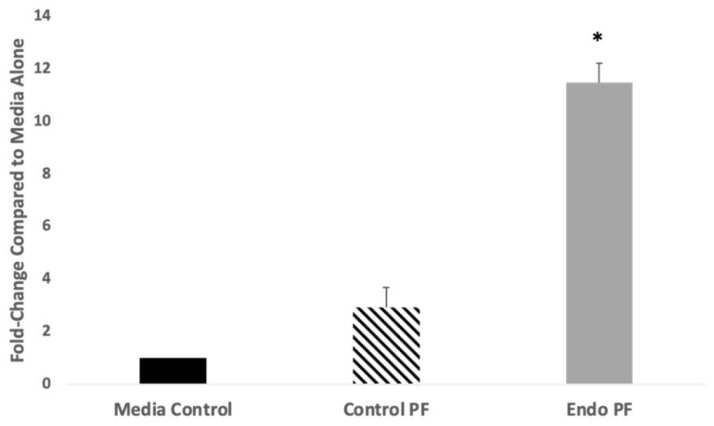
mRNA expression of PHF19 in endometrial cells treated with PF. When Ishikawa endometrial cells were treated with endo PF, expression of *PHF19* was shown to increase 11.50-fold relative to media alone treated cells (*p* = 0.0474). * *p* < 0.05 compared to media alone.

The polycomb-like proteins, PHF1 and PHF19 are critical components of PRC2 complex, both of which showed enrichment after EZH2 IP in the endo PF treated cells (fold change, 13.31 and 4.76-fold, respectively). Both these proteins are shown to work with the PRC2 complex in the manner similar to JARID2 in which they form subcomplexes with PRC2 core components and modulate the enzymatic activity of PRC2 and its recruitment [52,53]. Interestingly, PHF19 is also shown to interact with miR-155 to bring the PRC2 complex to its target [54]. In order to validate the ChIP findings, mRNA expression of *PHF19* was determined in the PF treated endometrial cells using qPCR. mRNA analysis revealed that *PHF19* was upregulated in endometrial cells treated with both control and endo PF when compared to media alone cells with the expression of *PHF19* reaching almost 11.5-fold in the endo PF treated cells (*p* = 0.0474), suggesting that PHF19 may be working along with miR-155 to bring the PRC2 complex to its target and promote endometriosis (Figure 8). 

### 2.9. Promoter Methylation of Inflammatory Genes

To assess changes in promoter methylation patterns in PF treated cells, a global DNA methylation array of genes involved in inflammation and autoimmunity was performed. The heat map in Figure S2A presents a range (from 0 to 100) of “M”, the fraction of input genomic DNA containing 2+ methylated CpG sites in the targeted region of a gene. Genes that were shown to be impacted by DNA methylation by having significant *p*-values (<0.05) (Figure S2B) were C-C Motif Chemokine Ligand 25 (CCL25), Cluster of Differentiation 8a (CD8A), CCAAT Enhancer Binding Protein Beta (CEBPB), Dipeptidyl peptidase 4 (DPP4), forkhead box P3 (FOXP3), interleukin-4 receptor (IL4R), Jun Proto-Oncogene, AP-1 Transcription Factor Subunit (JUN), Mitogen-activated protein kinase 14 (MAPK14), MHC class I polypeptide-related sequence B (MICB), and transforming growth factor beta 1 (TGFB1). All genes had an increased methylation pattern in cells treated with endo PF compared to the media control. The exception was MAPK14, which decreased in methylation in the endo PF treated cells. FOXP3 M values were 54.02% in endo PF treated cells (*p* < 0.0001), 26.54% in control PF treated cells (*p* = 0.0151), and 0.23% in media control. Bisulfite sequencing will be used in the future to better understand the methylation patterns of sample DNA.

## 3. Discussion

Our laboratory has been studying mechanisms leading to endometriosis and pain experienced by endometriosis patients [55,56,57,58]. This study stemmed from our previous investigations into the miRNA profile of endometriosis tissues and PF treated cells [33]. Nineteen percent of differentially expressed miRNAs in endo tissues targeted JARID2. Despite the global downregulation seen in the micronome of endometriotic tissues [33], miRNAs that targeted JARID2 were highly expressed in the eutopic tissues of endometriosis patients who also experienced pain as a symptom. The overexpression of miR-148a, miR-29a [33], and miR-155 in endo tissues (Figure 1B) seemed to further support this theory. As shown in Figure 1A, there was an increased expression of PRC2 complex proteins such as *EED* (0.0067), as well as a noticeable trend in overexpression of corresponding genes in ectopic tissues from endometriosis patients, particularly in *EZH2*. This correlates with the findings of Colon-Caraballo and colleagues [8,23] and supports the characterization of EZH2 as a contributor to transcriptional repression and progression of the disease. 

Although miR-155 was not originally identified based on the micronome array (*p* > 0.05), its relationship with JARID2 has recently drawn the attention of researchers in the field of inflammatory diseases [34,35]. miR-155 seems to play a key intermediate that regulates the crosstalk between JARID2 and PRC2 complex. miR-155 also plays a role in inflammation by working with FOXP3 to promote an inflammatory environment, since it has been shown that FOXP3 induces miR-155 expression [38,42]. Hence, miR-155 is a potential therapeutic target. This study explored the role of miR-155 in endometriosis by studying its interactions with the PRC2 complex, JARID2 and FOXP3. Endometrial cells were transfected with a miR-155 mimic or antagonist and then exposed to endo or control PF treatments. All PRC2 complex proteins examined showed an increase in expression in endo PF treated cells when compared to media alone treated cells. However, the cells transfected with a miR-155 mimic showed a downregulation of PRC2 complex proteins when exposed to either control or endo PF. The effect of gain- or- loss- of function of miR-155 on *JARID2* expression was interesting. miR-155 mimic transfected cells treated with control PF showed an increase in *JARID2* expression, while endo PF showed no change in expression. When transfected with the miR-155 inhibitor, no statistical difference in expression was seen in cells treated with control or endo PF. These results were unexpected and suggest that the miR-155 regulation of JARID2 is not sufficient to alter its expression. Hence, other transcription factors and/or epigenetic mediators could play a role in its aberrant expression in endometriosis. 

FOXP3 showed a significant increase in expression in both control and endo PF treated cells when transfected with a miR-155 mimic (Figure 7C), which paralleled the results seen for mRNA expression (Figure 6C). Such interactions between miR-155 and FOXP3 has been observed earlier. In diffuse B-cell lymphoma (DLBCL), high FOXP3 expression was correlated with a poor prognosis in patients and when miR-155 was silenced in these cells, there was a parallel decrease in FOXP3 levels [59]. In breast cancer, it was found that FOXP3 and miR-155 work together to down regulate ZEB2, resulting in reduced invasion [42]. 

Methylation of the FOXP3 promoter could be partly responsible for pain that women with endometriosis may experience based on the trend of increased methylation in cells treated with PF from endo patients, particularly those reporting pain (Figure S2). This has been seen in both biliary atresia and prostatitis [60,61]. Bamidele and colleagues looked at the interaction of EZH2 and FOXP3 in inflammatory bowel disease and found that a mutation in FOXP3 disrupted EZH2 recruitment and its co-repressive function. They also showed that IL-6 voided the FOXP3-EZH2 interaction and that this destabilized interaction may drive the gastrointestinal inflammation [62]. This disruption in interaction may also be true in endometriosis, since we and others have shown that IL-6 is increased in patients with endometriosis [55,63]. While *FOXP3* mRNA expression in endo PF-treated cells trended to be higher than that of cells treated with control PF, there was no statistical significance observed between the two treatments. These results suggest that FOXP3 is working alongside miR-155 to modulate the expression of EZH2.

*EZH2* mRNA expression in cells treated with both control and endo PF were higher when compared to media alone cells, but there was no statistical difference seen. However, an upregulation was seen in H3K27me3 in cells treated with endo PF. This is also significant as H3K27me3 is the downstream target of EZH2 and performs the transcriptional repression in cells [64]. The benefit of studying the PRC2 complex proteins in tissues and treated cells gave us the ability to compare short-term (in vitro) and long-term (in vivo) effects of peritoneal fluid on endometrial cells. This difference is likely to contribute to explaining the disparities in the observed results. 

ChIP-qPCR was used to better understand the regulatory roles of JARID2 and EZH2 and their cross-interactions in endometriosis. By observing how it binds to regulatory elements of various genes, a sense of how the mechanisms described above differ between PF from patients with and without endometriosis was gained. The data presented in Table 1 showed that the pull-down expression of JARID2 by EZH2 IP was by over 5-fold higher in cells treated with endo PF compared to control PF. It is interesting to note that, while not significant, JARID2 IP has a fold-change greater than 1 for EZH1, while for EZH2 it is less than 1 for endo PF treated cells. This suggests that the JARID2 interaction with EZH2 may not be as strong as it is with EZH1, which can also methylate H3K27 to contribute to transcriptional repression. Although it is typically associated with active domains, EZH1 can actually achieve repressive results similar to EZH2 via additional histone modifications [65,66,67]. It is interesting to note that when comparing genes after immunoprecipitations by the two antibodies (JARID2 and EZH2) in the two PF (endo or control PF) treated cells (Table 1B), the endo PF treated cells showed a trend of having a fold-change less than 1 when comparing JARID2 vs. EZH2 IP. This suggests that EZH2 in endo PF treated cells is having more of an effect on the expression of all genes in the array (PRC2 complex core, alternate and binding partners) when compared to JARID2 further supporting a role for EZH2 in endometriosis. 

One gene that should be noted and that was shown to have higher fold-change post EZH2 IP in endo PF treated cells vs. control PF treated cells was PHF19. PHF19 is a gene silencer and co-factor that can bind H3K36me3, which allows it to act as a recruiter for the PCR2 complex [68,69]. PHF19 also promotes tumorigenesis by the enhancement of the deposition of H3K27me3 and when PHF19 is depleted, this led to a loss of H3K27me3 domains [70]. This suggests that another mechanism which may be at play in transcriptional repression involves PHF19. PHF19 has also been deemed to play a role in the switch from proliferative to invasive states in melanoma cells [71]. Thus there are studies suggesting targeting PHF19 as an alternate strategy to inhibit EZH2 [72]. This study found that *PHF19* mRNA expression was significantly upregulated in cells treated with endo PF (Figure 8). This may suggest that miR-155 and PHF19 may be working together to bring the PRC2 complex to its targets in endometriosis. Putting these results together with miR-155 transfection studies, this study suggests that while miR-155 and PHF19 may be the main helper in regulating the PRC2 complex in endometriosis, JARID2 may be taking up the slack when miR-155 is inhibited. 

**Figure 9:**
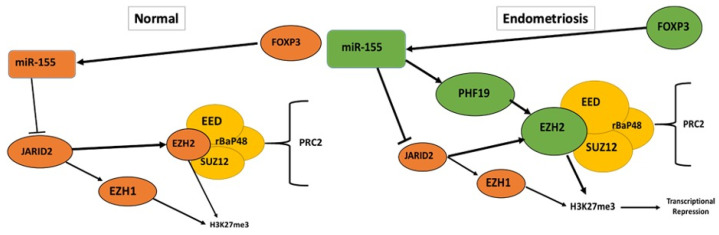
Proposed schematic of the epigenetic crosstalk playing a role in endometriosis. Mechanism proposed in normal women vs. women with endometriosis. Arrows indicate activation or general targeting while “T” bars indicate inhibition.

The findings presented here, as summarized in Figure 9, provide potential mechanisms that may be at play in endometriosis patients. This study shows that in the presence of endometrial PF, all the components of the PRC2 complex, along with JARID2, FOXP3 and miR-155 are increased in expression when compared to control PF. Gain-or loss-of function of miR-155 showed an effect on PRC2 complex proteins but not on JARID2 levels. This suggested that other epigenetic regulators may be involved. ChiP-qPCR pull-down studies using JARID2 or EZH2 antibodies in PF treated cells showed alterations in epigenetic proteins associated with either of these complexes. In addition to the known binding partners such as EZH1, DNMT3B etc, the expression of PHF19 (a PRC2 complex co-factor) was highly upregulated in EZH2 compared to JARID2 pull-down assay. This finding, in addition to what is known in the literature [54,69,70,72], it is presumable that in women with endometriosis, FOXP3/miR-155, in conjunction with PHF19, co-localizes with the PRC2 complex to promote its interaction and function with its targets. This leads to the increased H3K27me3 deposition thus modulating gene transcription. In contrast, this complex has a reverse effect on JARID2, thus preventing its association with the PRC2 complex, unless miR-155 is altered. This novel crosstalk among key epigenetic regulators leads to an increase in inflammation and growth of endometriotic lesions. This opens the door for testing newer targets in addition to the EZH2 inhibitors and miRNA mimics/antagonists currently being tested in endometriosis. For example, although histone demethylase inhibitors are thought to be ineffective against JARID2 due to its lack of true demethylase activity, additional investigations into the role of JARID2 in endometriosis could uncover alternate options to therapeutically regulate it, such as dihydroartemisinin which has been used in prostate cancer [73]. Additionally, the role for PHF19 as the master-regulator of the miR-155-PRC2 complex-JARID2 crosstalk is also a viable candidate for therapy and should be further explored in endometriosis. 

## 4. Materials and Methods

### 4.1. Human Subject Participants

Women ages 18 to 60 years, undergoing tubal ligation or having non-endometriosis disorders (controls, *n* = 12) or patients with endometriosis (“endo”, laparoscopically diagnosed followed by pathological confirmation and/or patients with symptoms, *n* = 14) were recruited from Obstetrics-Gynecology clinic at Cabell Huntington Hospital, Joan C Edwards School of Medicine, Marshall University, in Huntington, WV, USA. This HIPAA compliant study was approved by the Institutional Review Board of the Marshall University School of Medicine and was carried out per the principles of the Declaration of Helsinki. All patients were consented prior to the study. The inclusion criteria included women ages 18–60 years old, with normal menstrual cycles and otherwise in normal health (except for pain and endometriosis) who have not been on any hormonal medication for at least one month before sample collection. Exclusion criteria included subjects with current medical illnesses such as diabetes, cardiovascular disease, hyperlipidemia, hypertension, systemic lupus erythematosis or rheumatologic disease, positive HIV/AIDS, active infection. Subjects were asked to stop multivitamins that contain high levels of antioxidants and anti-inflammatory medications prior to sample collection. 

In this study, majority of the samples where from endo patients diagnosed with stage I/II and only one at stage IV. Pathological confirmation for endo patients classified the patients as mostly belonging to the peritoneal or uterine serosa pathology. All women completed a gynecologic/infertility history form, a pre-operative quality of life questionnaire and assessment of pain using a visual analog scale for assessment of endometriosis associated pain (dysmenorrhea, non-menstrual pelvic pain, dyspareunia, and dyschesia) (adapted from the validated International Pelvic Pain Society’s Pelvic Assessment Form). Date of their last menstrual period was used to assess their cycle time. 

### 4.2. RNA and Protein Isolation in Peritoneal Fluid-Treated Cells

Peritoneal fluid (PF) (devoid of blood contamination) was collected on ice from all women during laparoscopic surgery. Peritoneal fluid was spun at 2000× *g* to remove any cellular debris. The supernatant was used immediately for studies or stored in a −80 °C freezer for future use. To establish a cell model of the peritoneal environment, Ishikawa cells, a human (39-year-old woman) established endometrial epithelial cell line (Cat No: 99040201, Sigma-Aldrich, St. Louis, MO, USA), were cultured in T75 flasks in complete media (DMEM/F12, 10% FBS, 1% Pen/Strep, 1% l-glutamine). These cells were used because they express characteristics similar to those of mature endometrial epithelial cells [74,75,76]. Approximately 70% confluent cells were treated with 1% PF from patients for 48 h in a DMEM/F12 media containing 1% charcoal-stripped FBS. Patient peritoneal fluid (PF) groups were control PF (fluid from women without endometriosis) and endo PF (fluid from women with endometriosis). The concentrations of PF chosen were based on our previous published studies [33,55]. At the end of the 48-h treatment, cells were collected using Qiazol Lysis reagent (Cat No: 79306, Qiagen, Gaithersburg, MD, USA) and RNA was isolated using the Qiagen miRNeasy Mini Kit (Cat No: 217004, Qiagen, Gaithersburg, MD, USA). The quantity and quality of RNA were measured in the NanoDrop 2000 spectrophotometer. Cell lysates for measuring proteins, were prepared in RIPA buffer containing protease inhibitors (Cat No: P2714, Sigma-Aldrich, St. Louis, MO, USA) and protein concentrations were measured using a modified Lowry protocol [77]. 

### 4.3. Endometrial Tissue Collection and RNA/Protein Isolation

Endometrial (eutopic) tissues from control patients (EuN), eutopic tissues from endometriosis (peritoneal endometriosis, “endo”) patients (EuE), and ectopic endometriotic tissues (EcE) from endo patients were removed during laparoscopy/laparotomy by a qualified physician. Biopsy fragments were immediately placed in RNA*later* solution (Cat No: 76104, Qiagen, Gaithersburg, MD, USA) and subsequently stored in a freezer at −80 °C. RNA extraction from 100 mg of tissue (eutopic and ectopic) was carried out using Qiazol Lysis Reagent (Cat No: 79306, Qiagen, Gaithersburg, MD, USA). Tissues were homogenized using zirconium oxide beads in a Bullet Blender^®^ homogenizer (SKU: BBX24, Next Advance, Troy, NY, USA) and RNA was isolated using the Qiagen miRNeasy Mini Kit following the manufacturer’s recommendations (Cat No: 217004, Qiagen, Gaithersburg, MD, USA). The quantity and quality of RNA were measured using the NanoDrop 2000 spectrophotometer (Cat No: ND2000, Thermo Scientific, Waltham, MA, USA). Protein lysates from 50 mg of tissue was homogenized in RIPA buffer prior to protein estimation by a modified Lowry method [77].

### 4.4. mRNA and miRNA Expression in Tissues and PF-Treated Endometrial Cells

RNA (which includes miRNA) isolated from the tissues and treated cells were used. cDNA synthesis from 1 µg of each sample was performed using iScript cDNA synthesis kit (Cat No: 1708890, Biorad, Hercules, CA, USA). mRNA expression was analyzed in the cDNA samples using SYBR Green (Cat No: 1725270, Biorad, Hercules, CA, USA) and the primers listed in Table S1. 18S was used for normalization of mRNA expression. For determining miRNA expression, cDNA synthesis from 2 µg of each sample was performed using miScript II RT Kit (Cat No: 218161, Qiagen, Gaithersburg, MD, USA). Following cDNA synthesis, the expression of miR-29a, miR-148a, and miR-155 in tissues and PF-treated cells were determined using the appropriate Qiagen Primer Assay Kit, as per the manufacturer’s instructions. A primer assay for RNU6 was used as housekeeping for miRNA expression.

### 4.5. Protein Expression in PF-Treated Cells and Patient Tissues

Total protein was measured using a modified Lowry method. Protein (7 µg for cells and 5 µg for tissues) was run on the automated Western blotting system, WES [78] (Cat No: 004-600, Protein Simple, San Jose, CA, USA). The primary anti-rabbit antibodies for EZH2 (1:50, Cat No: 5426S), FOXP3 (1:25, Cat No: 12632S), JARID2 (1:50, Cat No: 13594S), and H3K27me3 (1:25, Cat No: 9733S) (Cell Signaling, Danvers, MA, USA), anti-rabbit β-actin (1:100, Cat no: 4970S, Cell Signaling, Danvers, MA, USA), and anti-rabbit H3 (1:100, Cat No: 39451, Active Motif, Carlsbad, CA, USA) were used to measure expression levels within the samples. HRP-conjugated rabbit secondary antibody provided in the WES kit was used. Plates (12-230 kDa and 25 capillary) were run using default settings and results analyzed using the Compass for WES software (Version 5.0.1). Band area given by the software was used and normalized to β-actin or H3. Results were expressed as a ratio in which media alone (for cell treatments) or EuN (for patient tissues) was considered to be 1. It is important to note that proteins examined using the automated Western Blotting system, WES, will have different expected molecular weights compared to traditional Western blotting, due to differences in technology. 

### 4.6. Cell Transfection with miR-155 Mimic/Inhibitor

Cells were transfected using SiPORT™ NeoFX ™ transfection agent (Cat No: AM4510, Ambion, Austin, TX, USA) as recommended by the manufacturer. In short, the SiPORT™ NeoFX ™ was diluted in Opti-MEM^®^ Reduced Serum Media (Cat No: 31985062, Invitrogen, Carlsbad, CA, USA) and incubated for 10 min at room temperature. miR-155 mimic (Pre-miR™), inhibitor (Anti-miR™), positive control (anti-let-7c) (Cat no: 4392431, Thermo Scientific, Waltham, MA, USA), and negative control (Negative control #1) (Cat No: AM17010, Thermo Scientific, Waltham, MA, USA) were diluted in cell media (DMEM/F12, 10% FBS, 1% Pen/Strep, 1% l-glutamine) to a final concentration of 30 nM and then combined with the transfection agent and incubated for 10 min at room temperature. Transfection mixtures were added to 6-well plates and overlaid with cell suspensions. Cells were then incubated for 24 h prior to treatment with peritoneal fluid from control and endometriosis patients, as previously described. Transfection efficiency was tested by collecting cells in Qiazol and assessing miRNA expression using the miR-155 primer assay. RNA was isolated using the miRNeasy Mini Kit following the manufacturer’s recommendations (Cat No: 217004, Qiagen, Gaithersburg, MD, USA). RT-qPCR was used (as previously described) to determine the expression of key downstream targets such as, JARID2 and FOXP3.

Western blots were performed in the traditional manner. Total protein was measured using a modified Lowry method. Protein (35 µg) was separated on a 4–20% Tris-HCl gradient gel (Cat No: 4561096, Biorad, Hercules, CA, USA) and transferred onto nitrocellulose membranes. After washing with Tris-buffered saline with Tween 20 (TBST), the membranes were blocked in 5% bovine serum albumin or 5% milk in TBST for 1 h, then incubated at 4 °C overnight with anti-rabbit antibody against JARID2 (Cat No: 13594S), FOXP3 (Cat No: 12632S), EZH2 (Cat No; 5426S), and H3K27me3 (Cat No: 9733S) (1:1000, Cell Signaling, Danvers, MA, USA) and anti-mouse against β-actin (1:4000, Cat No: A5316, Sigma-Aldrich, St. Louis, MO, USA). Anti-rabbit antibody against H4/H3 was diluted 1:20,000 (Cat No: 07-108, Sigma-Aldrich, St. Louis, MO, USA). Dilutions for primary antibodies varies from that used for WES due to the different methods used. The membranes were washed and incubated with HRP-linked anti-rabbit or anti-mouse secondary antibody (1:6000, Cat No: A6154, A4416, Sigma-Aldrich, St. Louis, MO, USA) for one hour at room temperature. After washing, membranes were developed in HRP Substrate (Cat No: WBKLS05000, Millipore, Temecula, CA, USA) and imaged using the ChemiDoc system (Cat No: 1708265, Biorad, Hercules, CA, USA). Densitometric levels of protein bands were quantified and normalized to β-actin or H3. Results were expressed as a ratio in which media alone was 1. 

### 4.7. EpiTect Methylation Array 

An EpiTect Methyl II Complete PCR Array (Cat No: 335005, Qiagen, Gaithersburg, MD, USA) was used to examine the levels of methylation in genes involved in inflammation and autoimmunity, in PF treated Ishikawa endometrial cells. Cells were treated as previously described and collected. DNA was isolated from the cells and DNA quantity and quality of RNA were measured using the NanoDrop 2000 spectrophotometer (Cat No: ND2000, Thermo Scientific, Waltham, MA, USA). Protocol provided by the manufacturer was followed. Input DNA was obtained and aliquoted into four equal portions and subjected to mock, methylation sensitive, methylation-dependent, and double restriction endonuclease digestion. After digestion, the enzyme reactions were used for qPCR using Sybrgreen. Analysis was performed using algorithm provided by Qiagen/SA Biosciences (Gaithersburg, MA, USA). A heat map was created from the data provided using Prism software (Version 9.0.0) (GraphPad, Inc., La Jolla, CA, USA). 

### 4.8. Chromatin Immunoprecipitation (ChIP)

Chromatin Immunoprecipitation (ChIP) was performed using the Chromatrap ChIP-Seq kit (Cat No: 500189, Porvair, Ashland, VA, USA) using either JARID2 or EZH2 antibodies. Approximately 70% confluent Ishikawa cells were treated with 1% PF from patients for 48 h in a DMEM/F12 media containing 1% charcoal-stripped FBS. Proteins were cross-linked by adding formaldehyde (0.75% by volume) and allowing for a 10-min incubation at room temperature. Glycine (0.5M) was added and incubated for an additional 10 min. Cells were twice rinsed with PBS, collected in 1 mL PBS and pelleted by centrifugation. All other buffer and components used were obtained from the kit. Protocol v1.5 of the manufacturer’s instructions was followed (Porvair, Ashland, VA, USA). Cells were suspended in 800 µL of hypotonic buffer before being centrifuged and the nuclear pellet was separated and resuspended in 400 µL of pre-warmed lysis buffer. Sonication was performed using a Covaris ME220 (SKU: 500506, Woburn, MA, USA). Each sample was aliquoted into 3 different tubes prior to sonication. Each tube was sonicated for 100 s and sheering efficiency was verified using an agarose gel as a smear of DNA fragments between 100–500 bp in length. ChIP-grade anti-JARID2 antibody (Cat No: 13594, Cell Signaling, Danvers, MA, USA) and EZH2 (Cat No: 5246S, Cell Signaling, Danvers, MA, USA) was used for antibody precipitation. DNA concentration was determined by NanoDrop 2000 spectrophotometer. The Human Polycomb & Trithorax Complexes EpiTect ChIP qPCR Array (Cat No: 334211 GH-506A, Qiagen, Gaithersburg, MA, USA) consisting of primers for genes belonging to the polycomb and trithorax complexes (core, alternate, and additional components), as well as polycomb co-factors such as PHD finger protein 19 (PHF19) and heterochromatin (CBX) proteins was run for all samples. Percent enrichment and further statistical analysis was calculated using algorithm provided by Qiagen/SA Biosciences (Gaithersburg, MA, USA).

### 4.9. Statistical Analysis

Prism software (Version 9.0.0, GraphPad, Inc., La Jolla, CA, USA) was used for analysis of all the non-array qPCR and WES data obtained from human tissue and cell culture studies. All values were expressed as mean ± standard error of the mean (SEM). A one-way ANOVA followed by Tukey’s post hoc test was used to detect any significant *p*-values. *p* Values less than 0.05 were considered to be significant. 

## Figures and Tables

**Table 1:** ChIP-qPCR of PRC2 complex proteins in PF-treated cells. Chromatin Immunoprecipitation (ChIP) was used to analyze interactions between JARID2 and EZH2 and genes associated with the polycomb and trithorax complexes, normalized to IgG. **A**. Fold change values represent the ratio of enrichment/binding of JARID2 or EZH2 to various genes in endo PF-treated cells (*n* = 3) to enrichment in control PF treated cells (*n* = 3). Genes with a *p*-value < 0.05 are shown as bold and italicized. EZH1 and EZH2 are also shown but did not have a significant *p*-value for either JARID2 or EZH2 when comparing the two cell treatments. **B**. Fold change values representing the ratio of enrichment/binding in EZH2 precipitated cells (*n* = 3) to enrichment in JARID2 precipitated cells (*n* = 3) for both cell treatments. *p*-values < 0.05 are shown as bold and italicized along with EZH1, EZH2, and JARID2 which did not show significant *p*-values for either treatment.

**A**		**JARID2**		**EZH2**	
**Symbol**	**Gene Name**	**Fold Change (Endo PF/Control PF)**	***p*-Value**	**Fold Change (Endo PF/Control PF)**	***p*-Value**
ARID1A	AT rich interactive domain 1A (SWI-like)	3.39	*0.0117*	10.65	0.1342
ASXL2	Additional sex combs like 2 (*Drosophila*)	4.51	0.2999	8.88	*0.0395*
CXXC1	CXXC finger protein 1	3.70	*0.0139*	8.56	0.1014
DNMT3B	DNA (cytosine-5-) methyltransferase 3 beta	1.13	0.8213	4.25	*0.0165*
EZH1	Enhancer of zeste homolog 1 (*Drosophila*)	1.4713	0.7986	12.11	0.259
EZH2	Enhancer of zeste homolog 2 (*Drosophila*)	0.3789	0.5621	12.79	0.2746
INO80	INO80 homolog (*S. cerevisiae*)	1.50	0.6214	3.76	*0.0182*
JARID2	Jumonji, AT rich interactive domain 2	2.92	0.2653	5.05	*0.0498*
PHF1	PHD finger protein 1	4.55	*0.0316*	13.31	0.1494
PHF19	PHD finger protein 19	0.2890	0.2152	4.76	0.1622
**B**		**Control PF**		**Endo PF**	
**Symbol**	**Gene Name**	**Fold Change (JARID2/EZH2)**	***p*-Value**	**Fold Change (JARID2/EZH2)**	***p*-Value**
DNMT3B	DNA (cytosine-5-) methyltransferase 3 beta	2.49	*0.0164*	0.66	0.385
E2F6	E2F transcription factor 6	4.52	*0.00027*	0.51	0.4839
EZH1	Enhancer of zeste homolog 1 (*Drosophila*)	0.91	0.9143	0.34	0.3762
EZH2	Enhancer of zeste homolog 2 (*Drosophila*)	3.59	0.3551	0.68	0.6713
JARID2	Jumonji, AT rich interactive domain 2	1.6	0.6838	0.92	0.8731
MTF2	Metal response element binding transcription factor 2	15.63	*0.0234*	0.67	0.6671
PHF19	PHD finger protein 19	1.96	0.3848	0.12	0.1440
SMARCA5	SWI/SNF related, matrix associated, actin dependent regulator of chromatin, subfamily a, member 5	8.13	*0.039*	0.73	0.7004

## Data Availability

Data sharing not applicable.

## Supplementary Materials

The following are available online at https://www.mdpi.com/article/10.3390/ijms22073492/s1.

## References

[1] Burney R.O., Giudice L.C. (2012). Pathogenesis and pathophysiology of endometriosis. Fertil. Steril..

[2] Giudice L.C. (2010). Clinical practice. Endometriosis. N. Engl. J. Med..

[3] Ciarmela P., Critchley H., Christman G.M., Reis F.M. (2013). Pathogenesis of endometriosis and uterine fibroids. Obs. Gynecol. Int..

[4] Platteeuw L., D’Hooghe T. (2014). Novel agents for the medical treatment of endometriosis. Curr. Opin. Obstet. Gynecol..

[5] Rowlands I.J., Abbott J.A., Montgomery G.W., Hockey R., Rogers P., Mishra G.D. (2020). Prevalence and incidence of endometriosis in Australian women: A data linkage cohort study. BJOG.

[6] Guo S.W. (2009). Epigenetics of endometriosis. Mol. Hum. Reprod..

[7] Nasu K., Kawano Y., Tsukamoto Y., Takano M., Takai N., Li H., Furukawa Y., Abe W., Moriyama M., Narahara H. (2011). Aberrant DNA methylation status of endometriosis: Epigenetics as the pathogenesis, biomarker and therapeutic target. J. Obstet. Gynaecol. Res..

[8] Colon-Caraballo M., Monteiro J.B., Flores I. (2015). H3K27me3 is an Epigenetic Mark of Relevance in Endometriosis. Reprod. Sci..

[9] Stephens L., Whitehouse J., Polley M. (2013). Western herbal medicine, epigenetics, and endometriosis. J. Altern. Complem. Med..

[10] Brunty S., Mitcell B., Bou-Zgheib N., Santanam N. (2020). Endometriosis and ovarian cancer risk, an epigenetic connection. Ann. Transl. Med..

[11] Koninckx P., Kennedy S., Barlow D. (1999). Pathogenesis of endometriosis: The role of peritoneal fluid. Gynecol. Obs. Investig..

[12] Guo S.W. (2019). Genesis, genes and epigenetics of endometriosis-associated infertility. Nat. Rev. Endocrinol..

[13] Bird A. (2007). Perceptions of epigenetics. Nature.

[14] Deichmann U. (2016). Epigenetics: The origins and evolution of a fashionable topic. Dev. Biol..

[15] Steffen P.A., Ringrose L. (2014). What are memories made of? How Polycomb and Trithorax proteins mediate epigenetic memory. Nat. Rev. Mol. Cell Biol..

[16] Cao R., Wang L., Wang H., Xia L., Erdjument-Bromage H., Tempst P., Jones R.S., Zhang Y. (2002). Role of histone H3 lysine 27 methylation in Polycomb-group silencing. Science.

[17] Kuzmichev A., Nishioka K., Erdjument-Bromage H., Tempst P., Reinberg D. (2002). Histone methyltransferase activity associated with a human multiprotein complex containing the Enhancer of Zeste protein. Genes Dev..

[18] Geisler S.J., Paro R. (2015). Trithorax and Polycomb group-dependent regulation: A tale of opposing activities. Development.

[19] Fuks F. (2005). DNA methylation and histone modifications: Teaming up to silence genes. Curr. Opin. Genet. Dev..

[20] Vire E., Brenner C., Deplus R., Blanchon L., Fraga M., Didelot C., Morey L., Van Eynde A., Bernard D., Vanderwinden J.M. (2006). The Polycomb group protein EZH2 directly controls DNA methylation. Nature.

[21] Kondo Y. (2009). Epigenetic cross-talk between DNA methylation and histone modifications in human cancers. Yonsei Med. J..

[22] Colon-Caraballo M., Torres-Reveron A., Soto-Vargas J.L., Young S.L., Lessey B., Mendoza A., Urrutia R., Flores I. (2018). Effects of histone methyltrasnferase inhibition in endometriosis. Biol. Reprod..

[23] Seguinot-Tarafa I., Luna N., Suarez E., Appleyard C.B., Flores I. (2020). Inhibition of Histone Methyltransferase EZH2 Suppresses Endometriotic Vesicle Development in a Rat Model of Endometriosis. Reprod. Sci..

[24] Arosh J.A., Lee J., Starzinski-Powitz A., Banu S.K. (2015). Selective inhibition of prostaglandin E2 receptors EP2 and EP4 modulates DNA methylation and histone modification machinery proteins in human endometriotic cells. Mol. Cell. Endocrinol..

[25] Li G., Margueron R., Ku M., Chambon P., Bernstein B.E., Reinberg D. (2010). Jarid2 and PRC2, partners in regulating gene expression. Genes Dev..

[26] Klose R.J., Kallin E.M., Zhang Y. (2006). JmjC-domain-containing proteins and histone demethylation. Nat. Rev. Genet..

[27] Kooistra S.M., Helin K. (2012). Molecular mechanisms and potential functions of histone demethylases. Nat. Rev. Mol. Cell Biol..

[28] Dong H., Liu S., Zhang X., Chen S., Kang L., Chen Y., Ma S., Fu X., Liu Y., Zhang H. (2019). An Allosteric PRC2 Inhibitor Targeting EED Suppresses Tumor Progression by Modulating the Immune Response. Cancer Res..

[29] Sanulli S., Justin N., Teissandier A., Ancelin K., Portoso M., Caron M., Michaud A., Lombard B., Da Rocha S.T., Offer J. (2015). Jarid2 Methylation via the PRC2 Complex Regulates H3K27me3 Deposition during Cell Differentiation. Mol. Cell.

[30] Pasini D., Cloos P.A., Walfridsson J., Olsson L., Bukowski J.P., Johansen J.V., Bak M., Tommerup N., Rappsilber J., Helin K. (2010). JARID2 regulates binding of the Polycomb repressive complex 2 to target genes in ES cells. Nature.

[31] Landeira D., Fisher A.G. (2011). Inactive yet indispensable: The tale of Jarid2. Trends Cell Biol..

[32] Niu Y., DesMarais T.L., Tong Z., Yao Y., Costa M. (2015). Oxidative stress alters global histone modification and DNA methylation. Free Radic. Biol. Med..

[33] Wright K.R., Mitchell B., Santanam N. (2017). Redox regulation of microRNAs in endometriosis-associated pain. Redox Biol..

[34] Palma C.A., Al Sheikha D., Lim T.K., Bryant A., Vu T.T., Jayaswal V., Ma D.D. (2014). MicroRNA-155 as an inducer of apoptosis and cell differentiation in Acute Myeloid Leukaemia. Mol. Cancer.

[35] Escobar T.M., Kanellopoulou C., Kugler D.G., Kilaru G., Nguyen C.K., Nagarajan V., Bhairavabhotla R.K., Northrup D., Zahr R., Burr P. (2014). miR-155 activates cytokine gene expression in Th17 cells by regulating the DNA-binding protein Jarid2 to relieve polycomb-mediated repression. Immunity.

[36] Yao Y., Li G., Wu J., Zhang X., Wang J. (2015). Inflammatory response of macrophages cultured with Helicobacter pylori strains was regulated by miR-155. Int. J. Clin. Exp. Pathol..

[37] Jablonski K.A., Gaudet A.D., Amici S.A., Popovich P.G., Guerau-de-Arellano M. (2016). Control of the Inflammatory Macrophage Transcriptional Signature by miR-155. PLoS ONE.

[38] Kohlhaas S., Garden O.A., Scudamore C., Turner M., Okkenhaug K., Vigorito E. (2009). Cutting edge: The Foxp3 target miR-155 contributes to the development of regulatory T cells. J. Immunol..

[39] Berbic M., Fraser I.S. (2011). Regulatory T cells and other leukocytes in the pathogenesis of endometriosis. J. Reprod. Immunol..

[40] Podgaec S., Rizzo L.V., Fernandes L.F., Baracat E.C., Abrao M.S. (2012). CD4(+) CD25(high) Foxp3(+) cells increased in the peritoneal fluid of patients with endometriosis. Am. J. Reprod. Immunol..

[41] Olkowska-Truchanowicz J., Bocian K., Maksym R.B., Bialoszewska A., Wlodarczyk D., Baranowski W., Zabek J., Korczak-Kowalska G., Malejczyk J. (2013). CD4(+) CD25(+) FOXP3(+) regulatory T cells in peripheral blood and peritoneal fluid of patients with endometriosis. Hum. Reprod..

[42] Brown C.Y., Dayan S., Wong S.W., Kaczmarek A., Hope C.M., Pederson S.M., Arnet V., Goodall G.J., Russell D., Sadlon T.J. (2018). FOXP3 and miR-155 cooperate to control the invasive potential of human breast cancer cells by down regulating ZEB2 independently of ZEB1. Oncotarget.

[43] Shen Z., Chen L., Yang X., Zhao Y., Pier E., Zhang X., Yang X., Xiong Y. (2013). Downregulation of Ezh2 methyltransferase by FOXP3: New insight of FOXP3 into chromatin remodeling?. Biochim. Biophys. Acta.

[44] Xiong Y., Khanna S., Grzenda A.L., Sarmento O.F., Svingen P.A., Lomberk G.A., Urrutia R.A., Faubion W.A. (2012). Polycomb antagonizes p300/CREB-binding protein-associated factor to silence FOXP3 in a Kruppel-like factor-dependent manner. J. Biol. Chem..

[45] Barcena de Arellano M.L., Mechsner S. (2014). The peritoneum—An important factor for pathogenesis and pain generation in endometriosis. J. Mol. Med..

[46] Carpinello O.J., Sundheimer L.W., Alford C.E., Taylor R.N., DeCherney A.H., Feingold K.R., Anawalt B., Boyce A., Chrousos G., de Herder W.W., Dungan K., Grossman A., Hershman J.M., Hofland H.J., Kaltsas G. (2000). Endometriosis. Endotext.

[47] Bedaiwy M.A., Falcone T., Sharma R.K., Goldberg J.M., Attaran M., Nelson D.R., Agarwal A. (2002). Prediction of endometriosis with serum and peritoneal fluid markers: A prospective controlled trial. Hum. Reprod..

[48] Jorgensen H., Hill A.S., Beste M.T., Kumar M.P., Chiswick E., Fedorcsak P., Isaacson K.B., Lauffenburger D.A., Griffith L.G., Qvigstad E. (2017). Peritoneal fluid cytokines related to endometriosis in patients evaluated for infertility. Fertil. Steril..

[49] Braza-Boils A., Salloum-Asfar S., Mari-Alexandre J., Arroyo A.B., Gonzalez-Conejero R., Barcelo-Molina M., Garcia-Oms J., Vicente V., Estelles A., Gilabert-Estelles J. (2015). Peritoneal fluid modifies the microRNA expression profile in endometrial and endometriotic cells from women with endometriosis. Hum. Reprod..

[50] Braza-Boils A., Gilabert-Estelles J., Ramon L.A., Gilabert J., Mari-Alexandre J., Chirivella M., Espana F., Estelles A. (2013). Peritoneal fluid reduces angiogenesis-related microRNA expression in cell cultures of endometrial and endometriotic tissues from women with endometriosis. PLoS ONE.

[51] Bitler B.G., Aird K.A., Zhang R. (2016). Epigenetic synthetic lethality in ovarian clear cell carcinoma: EZH2 and ARID1A mutations. Mol. Cell. Oncol..

[52] Dong C., Nakagawa R., Oyama K., Yamamoto Y., Zhang W., Dong A., Li Y., Yoshimura Y., Kamiya H., Nakayama J.I. (2020). Structural basis for histone variant H3tK27me3 recognition by PHF1 and PHF19. Elife.

[53] Li H., Liefke R., Jiang J., Kurland J.V., Tian W., Deng P., Zhang W., He Q., Patel D.J., Bulyk M.L. (2017). Polycomb-like proteins link the PRC2 complex to CpG islands. Nature.

[54] Ji Y., Fioravanti J., Zhu W., Wang H., Wu T., Hu J., Lacey N.E., Gautam S., Le Gall J.B., Yang X. (2019). miR-155 harnesses Phf19 to potentiate cancer immunotherapy through epigenetic reprogramming of CD8(+) T cell fate. Nat. Commun..

[55] Ray K., Fahrmann J., Mitchell B., Paul D., King H., Crain C., Cook C., Golovko M., Brose S., Golovko S. (2015). Oxidation-sensitive nociception involved in endometriosis-associated pain. Pain.

[56] Ray K.L., Mitchell B.L., Santanam N. (2014). Power over pain: A brief review of current and novel interventions for endometriosis-associated pain. J. Endometr. Pelvic Pain Disord..

[57] Rong R., Ramachandran S., Santanam N., Murphy A.A., Parthasarathy S. (2002). Induction of monocyte chemotactic protein-1 in peritoneal mesothelial and endometrial cells by oxidized low-density lipoprotein and peritoneal fluid from women with endometriosis. Fertil. Steril..

[58] Santanam N., Kavtaradze N., Murphy A., Dominguez C., Parthasarathy S. (2013). Antioxidant supplementation reduces endometriosis-related pelvic pain in humans. Transl. Res..

[59] Zhang J., Wei B., Hu H., Liu F., Tu Y., Zhao M., Wu N. (2017). Preliminary study on decreasing the expression of FOXP3 with miR-155 to inhibit diffuse large B-cell lymphoma. Oncol. Lett..

[60] Li K., Zhang X., Yang L., Wang X.X., Yang D.H., Cao G.Q., Li S., Mao Y.Z., Tang S.T. (2016). Foxp3 promoter methylation impairs suppressive function of regulatory T cells in biliary atresia. Am. J. Physiol. Gastrointest. Liver Physiol..

[61] Chen J., Zhan C., Zhang L., Zhang L., Liu Y., Zhang Y., Du H., Liang C., Chen X. (2019). The Hypermethylation of Foxp3 Promoter Impairs the Function of Treg Cells in EAP. Inflammation.

[62] Bamidele A.O., Svingen P.A., Sagstetter M.R., Sarmento O.F., Gonzalez M., Neto M.B.B., Kugathasan S., Lomberk G., Urrutia R.A., Faubion W.A. (2019). Disruption of FOXP3-EZH2 Interaction Represents a Pathobiological Mechanism in Intestinal Inflammation. Cell. Mol. Gastroenterol..

[63] Li S., Fu X., Wu T., Yang L., Hu C., Wu R. (2017). Role of Interleukin-6 and Its Receptor in Endometriosis. Med. Sci. Monit..

[64] Muller J., Hart C.M., Francis N.J., Vargas M.L., Sengupta A., Wild B., Miller E.L., O’Connor M.B., Kingston R.E., Simon J.A. (2002). Histone methyltransferase activity of a Drosophila Polycomb group repressor complex. Cell.

[65] Mochizuki-Kashio M., Aoyama K., Sashida G., Oshima M., Tomioka T., Muto T., Wang C., Iwama A. (2015). Ezh2 loss in hematopoietic stem cells predisposes mice to develop heterogeneous malignancies in an Ezh1-dependent manner. Blood.

[66] Shen X., Liu Y., Hsu Y.J., Fujiwara Y., Kim J., Mao X., Yuan G.C., Orkin S.H. (2008). EZH1 mediates methylation on histone H3 lysine 27 and complements EZH2 in maintaining stem cell identity and executing pluripotency. Mol. Cell.

[67] Son J., Shen S.S., Margueron R., Reinberg D. (2013). Nucleosome-binding activities within JARID2 and EZH1 regulate the function of PRC2 on chromatin. Genes Dev..

[68] Qin S., Guo Y., Xu C., Bian C., Fu M., Gong S., Min J. (2013). Tudor domains of the PRC2 components PHF1 and PHF19 selectively bind to histone H3K36me3. Biochem. Biophys. Res. Commun..

[69] Brien G.L., Gambero G., O’Connell D.J., Jerman E., Turner S.A., Egan C.M., Dunne E.J., Jurgens M.C., Wynne K., Piao L. (2012). Polycomb PHF19 binds H3K36me3 and recruits PRC2 and demethylase NO66 to embryonic stem cell genes during differentiation. Nat. Struct. Mol. Biol..

[70] Ren Z., Ahn J.H., Liu H., Tsai Y.H., Bhanu N.V., Koss B., Allison D.F., Ma A., Storey A.J., Wang P. (2019). PHF19 promotes multiple myeloma tumorigenicity through PRC2 activation and broad H3K27me3 domain formation. Blood.

[71] Ghislin S., Deshayes F., Middendorp S., Boggetto N., Alcaide-Loridan C. (2012). PHF19 and Akt control the switch between proliferative and invasive states in melanoma. Cell Cycle.

[72] Brien G.L., Jerman E., Campbell M., Adrian B.P. (2013). Abstract A28: Targeting PCL3/PHF19 as an alternative therapeutic strategy to EZH2 inhibition in PRC2-deregulated cancers. Cancer Res..

[73] Paccez J.D., Duncan K., Sekar D., Correa R.G., Wang Y., Gu X., Bashin M., Chibale K., Libermann T.A., Zerbini L.F. (2019). Dihydroartemisinin inhibits prostate cancer via JARID2/miR-7/miR-34a-dependent downregulation of Axl. Oncogenesis.

[74] Nishida M., Kasahara K., Kaneko M., Iwasaki H., Hayashi K. (1985). Establishment of a new human endometrial adenocarcinoma cell line, Ishikawa cells, containing estrogen and progesterone receptors. Nihon Sanka Fujinka Gakkai Zasshi.

[75] Bulun S.E., Cheng Y.H., Yin P., Imir G., Utsunomiya H., Attar E., Innes J., Julie Kim J. (2006). Progesterone resistance in endometriosis: Link to failure to metabolize estradiol. Mol. Cell. Endocrinol..

[76] Cho S., Mutlu L., Zhou Y., Taylor H.S. (2016). Aromatase inhibitor regulates let-7 expression and let-7f-induced cell migration in endometrial cells from women with endometriosis. Fertil. Steril..

[77] Lowry O.H., Rosebrough N.J., Farr A.L., Randall R.J. (1951). Protein measurement with the Folin phenol reagent. J. Biol. Chem..

[78] Harris V.M. (2015). Protein detection by Simple Western analysis. Methods Mol. Biol..

