# Supplementary material for: Peritoneal Modulators of EZH2-miR-155 Cross-Talk in Endometriosis
Source: Int J Mol Sci. 2021 Mar 28;22(7):3492. doi: 10.3390/ijms22073492 (PMC8038067; doi:10.3390/ijms22073492)
Supplement: Supplementary file 1 [file ijms-22-03492-s001.zip › suppl/ijms-1135665 suppl.pdf]

Supplementary Table 1. Primer sequences for genes used in the study. Primers were designed using NCBI GenBank and ordered from Invitrogen.

**Gene**	**Sequence**	
18S	F: GCAATTATTCCCCATGAACG	R: GGCCTCACTAAACCATCCAA
EED	F: CATTGGGCAATCAAGTTGGCA	R: ACAAGTGTGGAGAAAAAGCCTG
SUZ12	F: GTTACCGGTGAAGAAGCCGA	R: TTGGCTTCTCAAAGGCCTGG
EZH2	F: AAGGAGTTTGCTGCTGCTCT	R: ATTAATGGTGGGGGTGCTGG
JARID2	F: CTGCAGCACAAACGTGACTT	R: CATCAGCGAAACGTGAAGGTC
FOXP3	F: ACTGGGGTCTTCTCCCTCAA	R: GGGATTTGGGAAGGTGCAGA
PHF19	F: AATCCGTGGTCCCTATCCCA	R: ATAGGAGTCCCGAGTCCCTG

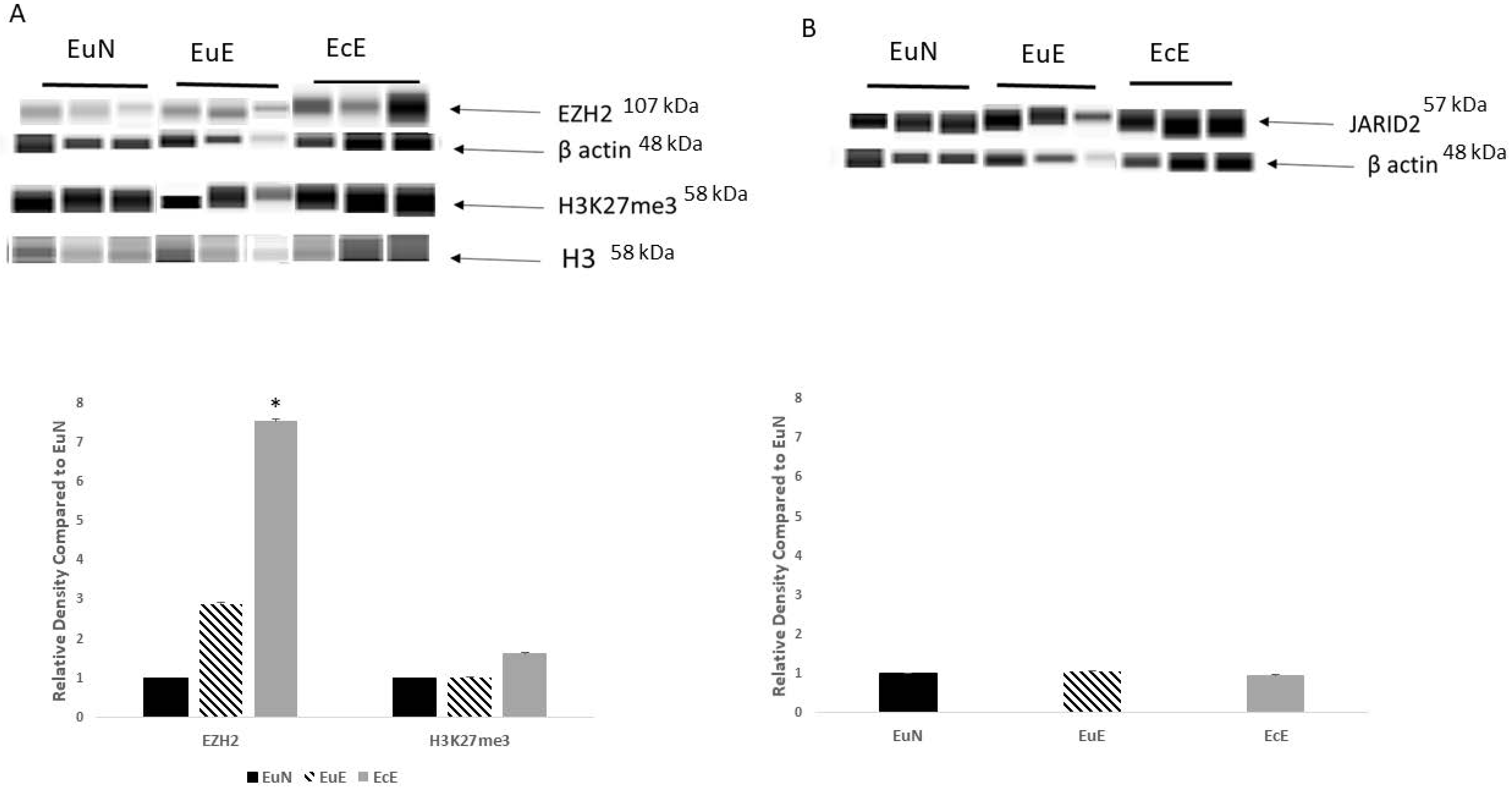

A
EuN
EuE
EcE
EZH2 107 kDa
β actin 48 kDa
H3K27me3 58 kDa
H3 58 kDa
Relative Density Compared to EuN
8
7
6
5
4
3
2
1
0
*
EZH2
H3K27me3
EuN
EuE
EcE
B
EuN
EuE
EcE
JARID2 57 kDa
β actin 48 kDa
Relative Density Compared to EuN
8
7
6
5
4
3
2
1
0
EuN
EuE
EcE

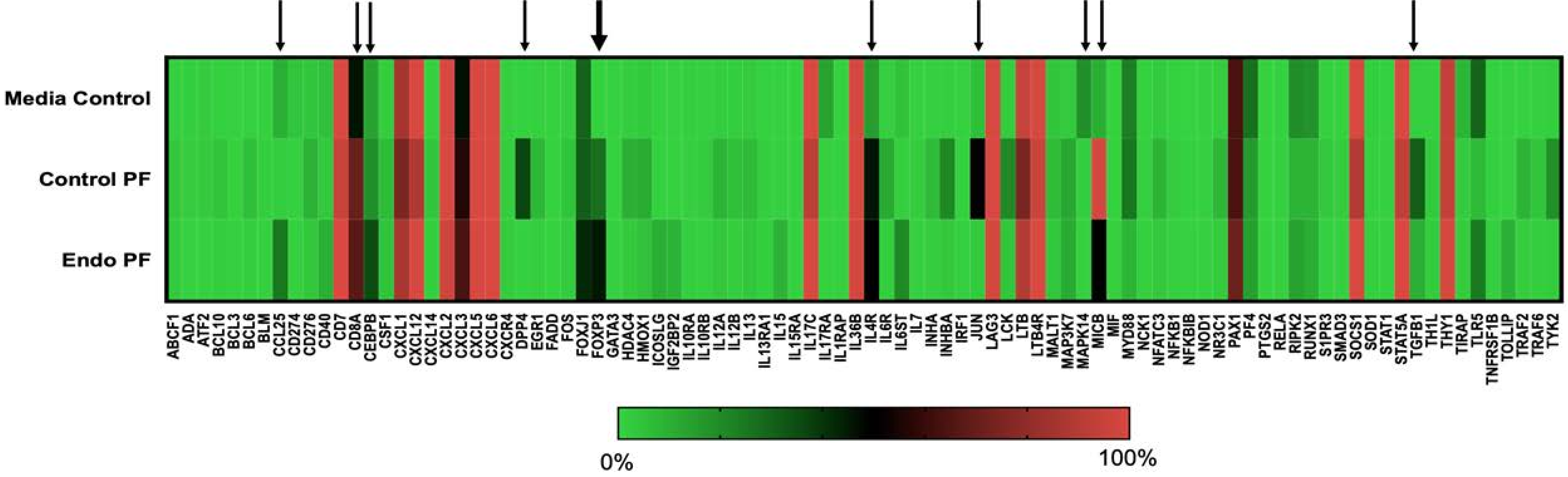

Gene Name	Media vs. Control	Media vs. Endo	Control vs. Endo
**CCL25**	0.6807	0.2104	**0.0222**
**CD8A**	**0.0297**	**0.0136**	0.9137
**CEBPB**	0.8816	**0.0448**	0.1575
**DPP4**	**0.0005**	0.9996	**<0.0001**
**FOXP3**	**0.0151**	**<0.0001**	0.0806
**IL4R**	0.0934	**0.0170**	0.9395
**JUN**	**0.0006**	0.9048	**<0.0001**
**MAPK14**	0.1639	**0.0403**	0.9970
**MICB**	**<0.0001**	**0.0043**	**0.0001**
**TGFB1**	**0.0052**	0.4354	**0.0403**
