# Supplementary material for: Peritoneal Modulators of EZH2-miR-155 Cross-Talk in Endometriosis
Source: Int J Mol Sci. 2021 Mar 28;22(7):3492. doi: 10.3390/ijms22073492 (PMC8038067; doi:10.3390/ijms22073492)
Supplement: Supplementary file 1 [file ijms-22-03492-s001.zip › suppl/suppl title.pdf]

Figure S1: Protein expression of EZH2, H3k27me3 and JARID2 in patient tissues. (**A**) WES images and densitometric analysis of protein bands of EZH2 and H3K27me3, in eutopic control (EuN), eutopic endo (EuE), and ectopic endo (EcE) tissues. A 7 fold increase in EZH2 expression was seen in EZH2 expression in EcE (p = 0.0219) compared to EuN; * $p<0.05$ (**B**) WES images and densitometric analysis of JARID2 protein expression, in eutopic control (EuN), eutopic endo (EuE), and ectopic endo (EcE) tissues. Protein expression was calculated relative to β-actin for EZH2 and JARID2; and H3 for H3K27me3. EuE and EcE were compared to EuN and shown as a ratio, where EuN was considered as 1.; **Figure S2**: Promoter Methylation patterns of inflammatory genes in PF-treated cells. (**A**) Heat map showing DNA methylation trends in PF-treated cells on promoters of genes associated with autoimmunity and inflammation. Treatment groups with green shades have lower methylation fractions than those with red shades. Arrows indicate where significant p-values were seen in genes which are shown in (**B**) *FOXP3*, is of interest, since it is tumor suppressor gene and known to regulate both miR-155 and EZH2. Endo PF: M = 54.02%, $p \leq 0.0001$; Control PF: M = 26.54%, p = 0.0151; Control media: M = 0.23%.; Table S1: Primer Sequences for genes used in the study.
